# Impact of Obstructive Sleep Apnea in Surgical Patients: A Systematic Review

**DOI:** 10.3390/jcm14145095

**Published:** 2025-07-17

**Authors:** Ioana-Medeea Titu, Damiana Maria Vulturar, Ana Florica Chis, Alexandru Oprea, Alexandru Manea, Doina Adina Todea

**Affiliations:** 1 Department of Surgery, Iuliu Hatieganu University of Medicine and Pharmacy, 400000 Cluj-Napoca, Romania; titu_ioana_medeea@elearn.umfcluj.ro (I.-M.T.);; 2 Department of Pneumology, Iuliu Hatieganu University of Medicine and Pharmacy, 400332 Cluj-Napoca, Romaniadtodea@umfcluj.ro (D.A.T.); 3 Thoracic Surgery Clinic, Leon Daniello Clinical Hospital of Pneumology, 400371 Cluj-Napoca, Romania; 4 Pneumology Clinic, Leon Daniello Clinical Hospital of Pneumology, 400371 Cluj-Napoca, Romania; 5 Cardiovascular Surgery Clinic, Niculae Stancioiu Heart Institute, 400001 Cluj-Napoca, Romania

**Keywords:** obstructive sleep apnea, perioperative complications, CPAP, preoperative screening

## Abstract

**Background/Objectives**: Obstructive sleep apnea is a prevalent, yet often underdiagnosed, condition characterized by recurrent upper airway obstruction during sleep, leading to significant perioperative risks in surgical patients. This systematic review aims to evaluate the incidence and impact of objectively diagnosed obstructive sleep apnea on postoperative outcomes across various surgical specialties—including bariatric, orthopedic, cardiac, and otorhinolaryngologic surgeries—and to assess the effectiveness of preoperative screening and perioperative management strategies. **Methods**: A comprehensive literature search of PubMed was conducted for studies published between January 2013 and December 2024, following Preferred Reporting Items for Systematic Reviews and Meta-Analyses guidelines. Included studies involved adult surgical patients with OSA confirmed by polysomnography or respiratory polygraphy. Studies were assessed for methodological quality using the Oxford Centre for Evidence-Based Medicine Levels of Evidence framework. **Results**: The findings consistently indicated that obstructive sleep apnea significantly increases the risk of postoperative complications, such as respiratory depression, atrial fibrillation, acute kidney injury, delirium, and prolonged hospital stay. Continuous positive airway pressure therapy demonstrated a protective effect in bariatric and cardiac surgeries, though its effectiveness in orthopedic and otorhinolaryngologic contexts was inconsistent, largely due to adherence variability and limited implementation. Preoperative screening tools such as the STOP-BANG questionnaire were widely used, but their utility depended on integration with confirmatory diagnostics. **Conclusions**: Obstructive sleep apnea represents a significant, modifiable risk factor in surgical populations. Preoperative identification and risk-adapted perioperative management, including CPAP therapy and multimodal analgesia, may substantially reduce postoperative morbidity. However, further randomized trials and cost-effectiveness studies are needed to optimize care pathways and ensure consistent implementation across surgical disciplines.

## 1. Introduction

Obstructive sleep apnea (OSA) is characterized by intermittent obstruction of the upper airway during sleep, leading to recurrent episodes of partial or complete cessation of breathing. This results in hypoxemia, hypercapnia, and frequent sleep arousals, which contribute to significant morbidity [1]. OSA is influenced by a range of risk factors, including obesity, male gender, advancing age, and certain conditions, such as lymphoreticular tissue hypertrophy of the pharynx and craniofacial abnormalities. These factors either reduce the patency of the upper airway during sleep or increase the propensity for airway collapse. Among these, obesity is the most significant risk factor, with OSA affecting 40% of adults with a body mass index (BMI) over 30, and 60% of individuals with metabolic syndrome [2].

The prevalence of OSA is notably high in the general population, and its incidence increases with age, reaching approximately 27% in women and 43% in men between the ages of 50 and 70, compared to 9% in women and 26% in men aged 30 to 49 [3]. Although OSA is very frequent in the general population, it also shows a high prevalence among surgical candidates [4]. OSA significantly impacts individual health and society by causing sleepiness, fatigue, cognitive impairments, mood disturbances, social withdrawal, and strained relationships [5]. It also negatively impacts workplace productivity, increases accident risk, and is linked to mental health conditions like depression and anxiety [6,7,8]. A 2016 Romanian study using the SAQLI (sleep apnea quality of life) questionnaire found significant impairments in daily functioning, emotional and social well-being, and symptoms, all of which improved markedly after three months of continuous positive airway pressure (CPAP) therapy, particularly among patients with COPD or morning headaches [9].

Several instruments are used for screening OSA, with varying degrees of effectiveness. The STOP-BANG questionnaire consistently demonstrates high sensitivity for detecting OSA [10], particularly in moderate-to-severe cases, where sensitivity ranges from 88% to 93% and reaches 97–100% for severe OSA [11]. The questionnaire’s effectiveness has been validated in a variety of populations, including the public, commercial drivers, and surgical patients [12]. The Berlin Questionnaire also exhibits good sensitivity and specificity for OSA screening in commercial vehicle drivers [13], while the NoSAS score and STOP-BANG are similarly effective in predicting OSA severity based on indices such as the apnea-hypopnea index (AHI) and oxygen desaturation index (ODI) [14]. In contrast, the Epworth Sleepiness Scale (ESS) shows inadequate sensitivity and specificity for OSA detection [15,16,17].

OSA diagnosis typically involves respiratory polygraphy (RP) or polysomnography (PSG), with PSG regarded as the gold standard. However, RP offers a simpler, more cost-effective alternative for uncomplicated cases [18]. PSG provides comprehensive data on sleep stages, respiratory patterns, and other physiological parameters [19], while RP focuses primarily on respiratory events [20]. OSA severity is classified using the AHI, with an AHI ≥ 5 indicating OSA and further categorized into mild (5–15), moderate (15–30), and severe (>30) based on the frequency of events per hour [21].

The prevalence of OSA among surgical patients is highly variable, with particularly elevated rates observed in high-risk populations, such as obese patients undergoing bariatric surgery [4]. Preoperative screening for OSA is essential, as undiagnosed OSA poses numerous risks, including increased postoperative complications such as cardiac and pulmonary events, oxygen desaturation, difficult intubation, and, in rare cases, mortality [22,23,24]. The perioperative management of patients with OSA is complex and requires careful consideration of factors such as anesthetic approach, airway management, and postoperative monitoring [25].

The American Society of Anesthesiologists Task Force has updated the Practice Guidelines for the Perioperative Management of Patients with OSA, emphasizing the importance of individualized care [26]. The guidelines advocate for the use of validated screening tools, such as the STOP-BANG questionnaire, preoperative risk stratification based on OSA severity and tailored perioperative management strategies to mitigate complications. Recommendations include avoiding sedative premedication due to the risk of airway obstruction, the use of regional anesthesia when feasible, and vigilant airway management during all phases of anesthesia. Postoperative care should include continuous monitoring and the use of CPAP in high-risk patients, along with early mobilization and opioid-sparing strategies.

OSA significantly contributes to the economic burden on healthcare systems and society, primarily due to its association with numerous medical conditions and non-medical consequences. A systematic cost-of-illness analysis in Italy estimated the annual societal costs attributable to OSA-related conditions to range between €10.7 and €32.0 billion. This considerable expense is mainly driven by direct healthcare costs, followed by indirect costs such as lost productivity due to morbidity and premature mortality. Furthermore, the quality-of-life losses due to undertreatment of moderate-to-severe OSA are substantial, highlighting the critical need for improved diagnostic and treatment pathways to mitigate these impacts [27].

This study aims to systematically review population-based studies to evaluate the evidence linking OSA with postoperative complications in patients undergoing bariatric, orthopedic, cardiovascular and otorhinolaryngology (ORL). While these areas have been relatively well-studied, the existing literature remains scarce regarding the association between OSA and postoperative outcomes in other surgical specialties, highlighting a critical gap in current perioperative risk assessment research.

The purpose of this review was to assess the current state of knowledge regarding the impact of OSA on postoperative outcomes and to provide a comparative overview of the role of OSA identification and management across different surgical specialties. The intention was not to formulate practice recommendations, as the available evidence remains limited and heterogeneous. Rather, the purpose was to synthesize existing data to highlight prevailing trends, knowledge gaps, and potential directions for future research in this evolving field, while also supporting the development of preliminary conclusions relevant to current clinical practice.

## 2. Materials and Methods

### 2.1. Literature Research

**Figure 1:**
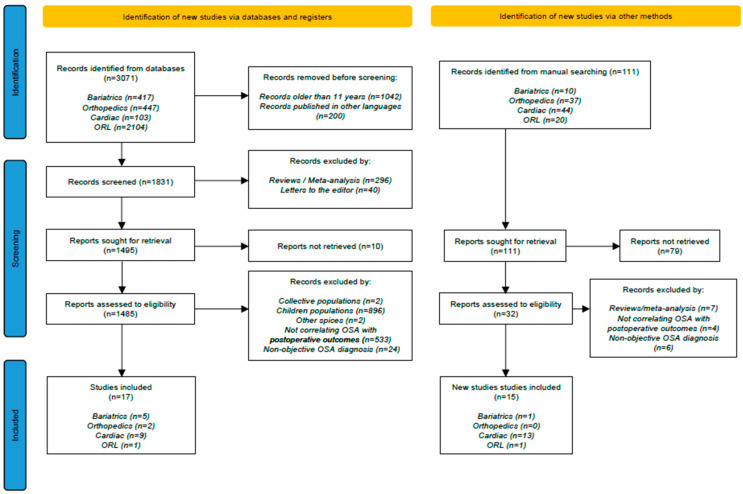
PRISMA Flow Diagram of Study Selection Process.

A systematic literature search was conducted in accordance with the Preferred Reporting Items for Systematic Reviews and Meta-Analyses (PRISMA) guidelines [28] to ensure transparency and reproducibility of the review process, as detailed in Figure 1. The research focused on articles describing postoperative complications in patients with OSA undergoing various types of surgery. The PubMed database was searched for articles published between January 2013 and December 2024, with a secondary manual search conducted to identify additional relevant studies that might have been overlooked initially. Grey literature (e.g., theses, dissertations, and conference proceedings) was excluded, as the review prioritized peer-reviewed, published studies.

The following Medical Subject Headings (MeSH) terms were used to refine the search: [“Sleep Apnea, Obstructive” AND “Bariatric Surgery”], [“Sleep Apnea, Obstructive” AND “Orthopedic Procedures”], [“Sleep Apnea, Obstructive” AND “Cardiac Surgical Procedure”] and [“Sleep Apnea, Obstructive” AND “Otorhinolaryngologic Surgical Procedures”]. To ensure that relevant studies were not excluded due to minor variations in terminology, both free-text searches and MeSH terms were employed, enhancing the search sensitivity.

The search retrieved 3071 unique citations from the PubMed database. After applying predefined inclusion and exclusion criteria, 17 articles were selected. This 11-year timeframe was intentionally chosen to reflect the most clinically relevant and methodologically consistent body of literature, anchored by the pivotal update to the American Society of Anesthesiologists (ASA) practice guidelines for the perioperative management of OSA, approved in late 2013. These guidelines marked a significant paradigm shift in perioperative screening, risk stratification, and management practices. By initiating the review from this point, the study captures the ensuing body of research shaped by these contemporary standards, while avoiding the heterogeneity and methodological limitations associated with older studies. Studies including clinical diagnoses of OSA were intentionally incorporated to emphasize the variability and gaps in current clinical practice, highlighting the need for consistent use of standardized screening and diagnostic protocols such as PSG or RP. Additionally, the manual search identified 111 additional studies, of which 15 met the eligibility criteria for inclusion in the analysis.

Due to substantial heterogeneity in study designs, populations, and outcome definitions, no meta-analyses or pooled estimates were generated. As a result, formal sensitivity analyses, reporting bias assessments, and certainty of evidence evaluations were not conducted. Instead, heterogeneity was explored qualitatively by grouping studies by surgical specialty and examining outcome variations in relation to factors such as OSA severity, comorbidities, anesthesia type, CPAP use, and postoperative monitoring. These findings should be interpreted with caution, considering the synthesis approach and potential for residual confounding.

### 2.2. Study Selection Criteria

The inclusion and exclusion criteria were mentioned in Table 1. Two independent reviewers screened titles and abstracts, assessed full-text articles for eligibility, and extracted data. Disagreements were resolved by consulting a third reviewer. Extracted data included study design, sample size, surgical type, OSA diagnostic method, effect size, effect type, perioperative outcomes, and statistical significance.

### 2.3. Synthesis Methods

Studies were grouped and synthesized by surgical specialty (bariatric, orthopedic, cardiac, and otorhinolaryngologic) to enable structured comparison. Extracted data were tabulated to facilitate thematic analysis of trends in postoperative complications among OSA patients. No quantitative meta-analysis was conducted due to substantial heterogeneity in study designs, outcome definitions, and patient populations.

### 2.4. Risk of Bias and Reporting Bias Assessment

Methodological quality was assessed using the Oxford Centre for Evidence-Based Medicine (OCEBM) Levels of Evidence (version 2009), which provides a structured and transparent framework for ranking evidence based on study design and methodological rigor [29]. The included studies were categorized into levels ranging from Level 1 (systematic reviews of randomized trials or high-quality randomized controlled trials) to Level 5 (expert opinion without explicit critical appraisal). This hierarchical approach allowed for a critical interpretation of the literature and supported the identification of findings with the highest reliability and clinical applicability.

Most of the studies in the bariatric, cardiac, orthopedic, and otorhinolaryngologic surgical domains were retrospective cohort studies, corresponding to Level 2b evidence with a Grade B recommendation. Only a few randomized controlled trials reached Level 1b, highlighting the relative paucity of high-level interventional research in this area. The application of OCEBM enabled a nuanced evaluation of study outcomes, distinguishing robust findings from those requiring cautious interpretation in clinical practice.

As this framework does not provide domain-based risk of bias assessment, no formal tools were used. Similarly, no assessments for reporting bias were conducted, as no meta-analyses were performed. Two reviewers independently applied OCEBM levels, resolving disagreements by addressing a third reviewer.

Overall, the integration of OCEBM contributes to the methodological transparency, credibility, and relevance of this review to evidence-based perioperative management of patients with OSA.

### 2.5. Certainty Assessment

No formal certainty assessment was performed due to the heterogeneity of the review. While OCEBM levels provided insight into study quality, they do not reflect certainty in outcome-specific evidence and should be interpreted accordingly.

## 3. Results

### 3.1. Perioperative Risks and Outcomes of OSA in Bariatric Surgery

To assess the correlation between OSA and postoperative complications following bariatric surgery, six publications were examined.

Zaremba et al. conducted a randomized crossover trial demonstrating that postoperative CPAP significantly reduced the AHI by 69% in morbidly obese patients and mitigated opioid-induced respiratory depression without causing hemodynamic instability [30]. Kong et al., in a retrospective analysis of 352 patients, found a significantly higher rate of pulmonary complications in untreated OSA patients compared to those receiving perioperative CPAP (14.9% vs. 2.95%, *p* = 0.0002) [31]. Goucham et al. further corroborated the safety of CPAP in severe OSA patients, reporting zero re-intubations or deaths, and suggesting ICU admission may not be necessary with proper monitoring [32].

O’Reilly et al. found no significant differences in postoperative cardiopulmonary complications or length of stay between screened and unscreened patients, challenging the universal necessity of preoperative sleep studies for asymptomatic individuals [33]. Increasing age, not OSA, was predictive of complications.

De Raaff et al. analyzed 277 patients with mild OSA and found no statistically significant difference in 30-day cardiopulmonary complication rates between those with and without positional OSA (POSA), defined by a supine AHI at least double that of lateral positions, suggesting CPAP may not be required in this subgroup [34].

Mokhlesi et al., using the Nationwide Inpatient Sample of 91,028 patients, revealed paradoxical findings [35]. While patients with sleep-disordered breathing exhibited higher rates of respiratory complications (e.g., emergent intubation OR 4.35), they also had lower in-hospital mortality (OR 0.34) and shorter lengths of stay (mean reduction: 0.25 days) [35].

Table 2 encompasses the characteristics of these studies pertaining to bariatric surgeries.

### 3.2. Perioperative Risks and Outcomes of OSA in Orthopedic Surgery

This synthesis integrates findings from two studies evaluating perioperative strategies in patients with OSA undergoing orthopedic procedures.

Wong et al. conducted a multicenter randomized controlled trial on 234 patients aged ≥ 60 years with newly diagnosed OSA undergoing elective hip or knee arthroplasty [36]. The incidence of postoperative delirium was low (2.7%) and did not significantly differ between those receiving auto-titrating CPAP therapy (0.9%) and controls (4.4%). No differences were observed in LOS or other complications [36].

Bai et al. investigated the safety of low-dose intrathecal morphine in 1326 patients with suspected or diagnosed OSA undergoing joint arthroplasty [37]. Pulmonary complications occurred in 1.3% of patients who received intrathecal morphine compared to 2.1% in the control group. After adjustment, intrathecal morphine use was not associated with increased risk of pulmonary complications [37].

Table 3 outlines the features of studies related to orthopedic operations.

### 3.3. Perioperative Risks and Outcomes of OSA in Cardiac Surgery

Twenty-two papers were analyzed to evaluate the association between OSA and postoperative complications after cardiac surgical or interventional procedures. The evidence firmly indicates a correlation between OSA and heightened postoperative morbidity across multiple outcomes.

Postoperative atrial fibrillation (POAF) was the most frequently reported complication associated with OSA [38,39,40,41]. The study published by van Oosten et al. demonstrated a progressive rise in POAF incidence with increasing OSA severity, from 29.7% in low-risk individuals to 51.4% in those with confirmed OSA (odds ratio [OR] = 2.18) [38]. Further, Fein et al. found that adherence to preoperative CPAP therapy significantly reduced the risk of POAF recurrence, with an atrial fibrillation-free survival of 71.9% among compliant patients compared to 36.7% in non-compliant counterparts [39].

In patients with rheumatic valvular heart disease undergoing cardiac valve replacement (CVR), Ding et al. reported that OSA was independently associated with prolonged ICU stay, increased duration of mechanical ventilation, and a higher incidence of postoperative pacemaker implantation [42].

OSA was further linked to heightened neurocognitive risk. Roggenbach et al. found that an AHI ≥ 19 conferred a 6.4-fold increased likelihood of postoperative delirium following elective cardiac surgery [43]. Delirious patients experienced prolonged ICU stay and required more intraoperative transfusions [43]. Additionally, two independent studies identified OSA as a significant predictor of acute kidney injury (AKI) post-CABG, reinforcing the systemic impact of sleep-disordered breathing [44,45].

Respiratory instability post-cardiac surgery, characterized by prolonged mechanical ventilation and vasopressor dependency, was significantly more common in OSA patients. Studies by Wolf et al. and Tefelmaier documented longer hospitalizations and increased postoperative respiratory complications [46,47]. Moreover, Rupprech et al. reported that moderate-to-severe sleep-disordered breathing was associated with increased rates of sepsis and an elevated 30-day mortality (OR = 10.1) [48].

OSA significantly increased the incidence of major adverse cardiovascular and cerebrovascular events (MACCEs). In peripheral arterial disease patients undergoing surgical revascularization, Utriainen et al. observed a 5-fold increase in MACCE risk (hazard ratio [HR] = 5.1) among those with AHI ≥ 20 [49]. Similarly, multicenter analyses revealed higher rates of myocardial infarction, unstable angina, and repeat revascularization procedures in OSA populations [40,50]. Teo et al. additionally demonstrated that impaired cardiac repolarization dynamics in OSA patients independently predicted MACCE occurrence [51]. A large international cohort found a MACCE incidence of 18.9% in OSA patients versus 14.0% in controls (adjusted HR = 1.57; *p* = 0.013) [52].

OSA was associated with increased healthcare utilization. Zhao et al. identified a 4.6-fold elevated risk of cardiovascular readmissions within 6 months following CABG among OSA patients [41]. Furthermore, Gao et al. established high-sensitivity C-reactive protein (Hs-CRP) as a biomarker correlating with OSA severity, POAF development, and extended hospitalization [53].

The influence of OSA extended to patients undergoing pulmonary vein isolation (PVI) for the treatment of AF. In a randomized controlled trial, CPAP use significantly reduced AHI but did not decrease AF recurrence post-ablation [54]. However, observational data from Szymanski et al. showed a direct relationship between OSA severity and AF recurrence following ablation [55]. Neilan et al. provided further mechanistic insights, linking OSA with adverse cardiac remodeling, including increased left atrial size and left ventricular mass, while demonstrating that CPAP therapy mitigated these structural changes and lowered the risk of recurrent AF [56].

Table 4 presents an overview of the key characteristics of the studies included in the analysis related to cardiac surgeries.

### 3.4. Perioperative Risks and Outcomes of OSA in Otorhinolaryngologic Surgery

Two clinical studies were analyzed to evaluate the impact of OSA on postoperative outcomes in otorhinolaryngologic surgeries. In the study by Passeri et al., 28 patients with OSA undergoing maxillomandibular advancement (MMA) experienced a significantly higher rate of complications compared to 26 controls with dentofacial deformities (100% vs. 73%, *p* = 0.003), including a threefold increase in total complications and a tenfold increase in major complications such as infections requiring reoperation [59].

In a complementary study by Kandasamy et al., among 345 patients undergoing uvulopalatopharyngoplasty (UPPP), 28.1% experienced postoperative complications, with respiratory issues such as oxyhemoglobin desaturation being the most common (12.8%) [60]. Higher AHI and BMI were significantly associated with increased risk of postoperative oxygen requirement, especially in patients with AHI ≥ 22 and BMI ≥ 30 (OR = 3.48) [60].

Neither study reported perioperative mortality, but both indicated that OSA significantly elevates the risk of postoperative morbidity in surgical populations.

Table 5 provides a summary of the main characteristics of the five studies included in the analysis, focusing on otorhinolaryngologic surgeries.

## 4. Discussion

This systematic review comprehensively synthesized existing evidence regarding the perioperative implications of OSA across bariatric, orthopedic, cardiac, and otorhinolaryngologic surgical populations. We structured the discussion to address specific clinical questions, integrating findings across specialties and interpreting results relative to the existing literature. This approach enables a nuanced understanding of OSA’s multifaceted impact on surgical patients.

### 4.1. Is Obstructive Sleep Apnea a Risk Factor for the Surgical Patient?

OSA has been consistently identified as an independent risk factor for perioperative complications across diverse surgical specialties. In cardiac surgery, patients with OSA exhibited significantly higher rates of POAF [44,48,57], AKI [44,45], and respiratory complications [58], indicating greater vulnerability.

Similarly, in bariatric surgery populations, OSA was linked to increased risks of emergent intubation and POAF, although this did not correspond to higher mortality rates, likely due to intensified perioperative monitoring and care [35]. These findings support the value of preoperative screening, particularly in high-risk individuals. However, the absence of increased complications among asymptomatic, unscreened patients suggests that targeted screening may offer a more cost-effective approach [33].

In orthopedic surgery, Bai et al. reported no significant increase in pulmonary complications in patients with diagnosed or suspected OSA receiving low-dose intrathecal morphine during joint arthroplasty [37]. Their findings indicate that with standardized multimodal analgesia and vigilant monitoring, OSA does not independently elevate perioperative risk. This aligns with broader evidence suggesting that AHI severity alone may not reliably predict postoperative outcomes, particularly when management protocols are optimized.

In the otorhinolaryngologic field, studies by Passeri et al. and Kandasamy et al. further emphasize OSA’s impact on surgical risk [59,60]. Patients undergoing MMA with a diagnosis of OSA were notably older, had higher ASA scores, and more comorbidities, resulting in a significantly higher incidence of severe postoperative complications [59]. Infections requiring reoperation or readmission were especially prevalent, highlighting the importance of preoperative risk stratification in this subgroup.

### 4.2. Does Obstructive Sleep Apnea Have a Significant Incidence in Surgical Populations to Request a Preoperative Screening?

The consistently high prevalence of OSA in surgical populations strongly supports the need for routine preoperative screening, particularly in high-risk groups such as bariatric patients, where rates may reach as high as 91% [4,32,33]. This trend extends to orthopedic cohorts as well; Bai et al. [37] reported that over 53% of patients undergoing arthroplasty had confirmed OSA, a pattern that reflects the broader risk in obese and elderly populations [36].

OSA is frequently underdiagnosed despite its substantial presence. In cardiovascular surgery, approximately 50–75% of patients present with an AHI ≥ 5, and nearly half have moderate-to-severe OSA (AHI ≥ 15) [38,58].

In ORL surgery, Kandasamy et al. observed significant early postoperative respiratory complications—particularly oxyhemoglobin desaturation—among UPPP patients, with elevated AHI and BMI emerging as key predictors [60]. These findings are supported by other cohorts with clinically diagnosed OSA, indicating that systematic preoperative identification of OSA can improve risk stratification and inform postoperative monitoring strategies in this surgical population [61,62,63].

### 4.3. Does CPAP Have a Role in Postoperative Complications Incidence in Patients with Obstructive Sleep Apnea?

CPAP therapy has demonstrated potential in reducing postoperative complications in patients with OSA, though its effectiveness appears to vary across surgical specialties. It has been associated with reductions in AHI and opioid-induced respiratory depression [30], pulmonary complications [31], and even ICU admission rates among adherent patients [32]. However, its benefit is less evident in studies that primarily included mild or asymptomatic cases [33,34] or those based on administrative data, where outcomes often diverge from chart-reviewed studies [64,65].

In cardiac surgery, CPAP use correlates with fewer respiratory and cardiovascular complications, particularly a reduction in postoperative atrial fibrillation [39,55]. Nevertheless, randomized trials yield mixed results, likely due to inconsistent adherence to CPAP therapy and variability in the timing of CPAP initiation [54].

In orthopedic surgery, evidence remains inconclusive. The PODESA trial, for example, found no statistically significant reduction in postoperative delirium among elderly patients treated with CPAP, despite a numerically lower incidence (0.9% vs. 4.4%)—a result likely influenced by poor adherence, with average nightly CPAP use falling to 2.7 h by postoperative day three [36]. Similar discrepancies have emerged in studies employing non-objective diagnostic methods, such as administrative codes or screening tools like STOP-BANG, where CPAP use was not consistently associated with improved outcomes, and, in some cases, was linked to higher complication rates, potentially due to indication bias [66,67,68]. Additionally, multimodal analgesia strategies have proven more effective than opioid-heavy regimens in minimizing respiratory and gastrointestinal complications [69]. Yet, physiological disturbances such as oxygen desaturation [52,70,71,72] and hypercapnia [73] may still occur, highlighting the need for comprehensive perioperative protocols that integrate both respiratory support and optimized analgesia.

### 4.4. Are There Validated Protocols for the Perioperative Management of Surgical Patients with OSA?

Validated perioperative management protocols for patients with OSA are well-established and widely applied in clinical practice, with key contributions from organizations such as the Society of Anesthesia and Sleep Medicine (SASM), the American Society of Anesthesiologists (ASA), and joint efforts from the American Academy of Sleep Medicine (AASM) [26,74,75].

These guidelines and the reviewed literature emphasize thorough preoperative screening using tools like STOP-BANG, the Berlin Questionnaire, and the Epworth Sleepiness Scale to identify at-risk individuals, particularly in high-prevalence populations such as bariatric surgery candidates. Positive screening may necessitate further diagnostic evaluation with PSG, followed by risk stratification to guide adapted management strategies, including CPAP therapy and appropriate postoperative monitoring. Intraoperative care focuses on mitigating respiratory complications through short-acting anesthetics, regional techniques, and meticulous airway management. Postoperatively, continuation or initiation of CPAP is vital to prevent airway collapse, especially in severe cases or those experiencing complications, often necessitating ICU-level monitoring with pulse oximetry and capnography. Pain control strategies prioritize opioid-sparing methods, such as multimodal analgesia and regional blocks, to reduce the risk of respiratory depression. Finally, discharge planning centers on promoting CPAP adherence, patient education, and ensuring follow-up for sleep studies and ongoing symptom monitoring to support long-term outcomes.

### 4.5. What Is the Cost-Effectiveness of Managing OSA in Surgical Patients?

The economic analyses available indicate that effective perioperative management of OSA, particularly through CPAP, is highly cost-effective, significantly reducing perioperative complications, intensive care admissions, and hospital stay durations [35,76,77]. Bariatric and cardiac surgery patients notably demonstrated clear economic advantages from proactive OSA management, leading to considerable healthcare resource savings [39]. Early initiation of CPAP therapy in diagnosed patients significantly reduces the incidence of respiratory and cardiovascular complications, thereby mitigating the need for intensive and costly postoperative interventions [78,79]. Studies show elevated readmission rates in patients with OSA, often associated with arrhythmia and respiratory issues [41]. Untreated OSA increases recurrent revascularization, exacerbating long-term healthcare costs [50].

Unmanaged OSA in orthopedic surgical patients is linked to increased postoperative healthcare resource utilization, including more frequent admissions to ICUs and extended monitoring in PACUs [66,80,81,82]. This increases the risk of perioperative complications and strains postoperative care resources. Proactive, individualized approaches, such as multimodal, opioid-sparing analgesia, can significantly reduce clinical complications and hospital costs [69]. Implementing structured perioperative OSA protocols, including preoperative screening and PAP therapy in the PACU, has been shown to mitigate ICU transfers and improve respiratory outcomes in high-risk patients [69,78].

Surgical treatment for otorhinolaryngologic OSA has shown cost-effectiveness, particularly compared to CPAP therapy. Initial expenses are higher, but long-term healthcare costs are significantly reduced [83]. Nasal surgery improves CPAP compliance in patients with nasal obstruction, and septoplasty attains cost-effectiveness over extended periods [84]. In South Korea, UPPP demonstrated nearly six times greater cost-effectiveness than PAP, with a lower cost per quality-adjusted life year [85]. Surgical therapies are cost-effective for controlling OSA in suitable patients and should be integrated into personalized therapy regimens.

## 5. Limitations

This systematic review, while extensive, is subject to several limitations that warrant consideration. First, the heterogeneity of the included studies—in terms of surgical specialties, study designs, outcome definitions, and OSA diagnostic modalities—may limit the generalizability of the findings and impede direct comparisons across studies. Although efforts were made to stratify outcomes by surgical type, the variation in perioperative management strategies and institutional protocols introduces potential confounding variables that are difficult to control for the synthesis.

Second, the reliance on retrospective data in a substantial proportion of included studies introduces inherent biases, including selection and information bias, and limits causal inference. The underrepresentation of randomized controlled trials, particularly those evaluating perioperative interventions like CPAP therapy, diminishes the strength of evidence supporting certain clinical recommendations.

Third, compliance with CPAP therapy was inconsistently reported and poorly measured across many studies, making it difficult to ascertain its true protective impact on postoperative outcomes. Furthermore, studies that assessed CPAP effects often failed to distinguish between compliant and non-compliant users or accounted inadequately for OSA severity.

## 6. Conclusions

This systematic review underscores the significant perioperative risks associated with OSA across a spectrum of surgical disciplines. Evidence consistently demonstrates that OSA is an independent predictor of a wide range of postoperative complications, including cardiopulmonary events, delirium, acute kidney injury, and prolonged hospital stays. These risks were more pronounced in patients with moderate-to-severe OSA and in older adults with multiple comorbidities.

While preoperative screening tools like the STOP-BANG questionnaire remain valuable for identifying at-risk individuals, their predictive value is limited without confirmatory diagnostics. While CPAP therapy shows promise in mitigating perioperative risk, particularly in cardiac and bariatric surgery, its efficacy remains variable in orthopedic and otorhinolaryngologic contexts, partly due to poor adherence and limited perioperative implementation.

The findings highlight the importance of incorporating individualized, evidence-based perioperative protocols for patients with OSA, including structured screening, CPAP use, opioid-sparing analgesia, and enhanced monitoring. Nonetheless, variations in clinical practice and study methodology underline the need for further high-quality randomized trials and cost-effectiveness analyses. Ultimately, recognizing and managing OSA as a modifiable perioperative risk factor represents a critical step toward improving surgical outcomes and optimizing healthcare resource utilization.

## Figures and Tables

**Table 1:** Summary of inclusion and exclusion criteria used to select studies evaluating postoperative outcomes in adult surgical patients with suspected or confirmed OSA.

Inclusion and Exclusion Criteria
**Inclusion Criteria** Studies involving adult surgical patients (≥18 years) with confirmed OSA through RP or PSG. Studies published in English. Longitudinal study designs include retrospective and prospective cohort studies, as well as randomized controlled trials (RCTs) and randomized crossover trials.
**Exclusion Criteria** Studies with clinically suspected OSA. Studies involving pediatric patients or pregnant women. Articles published as abstracts, conference proceedings, book chapters, letters to the editor, reviews, or meta-analyses. Animal studies or studies involving in vitro models. Studies involving collective populations where outcomes specific to OSA patients were not clearly distinguishable. Studies that did not explicitly mention OSA or its correlation with postoperative outcomes.

**Table 2:** Characteristics of studies included in the analysis pertaining to bariatric surgeries (RCT = randomized crossover trial; RS = retrospective study; PS = prospective study).

Author	Design	Type of Surgery	Sample Size		OSA Diagnosis	Effect Size	Effect Type	Complications	Significance	Oxford Level of Evidence	Grade of Recommendation
			OSA	Non-OSA							
O’Reilly et al., 2020 [33]	RS	bariatric surgery	300	210	RP/PSG		Logistic regression	Aspiration, atelectasis, pneumonia, hypoxia, respiratory failure, atrial fibrillation, ICU admission, persistent significant OSA	Pulmonary complication: *p* = 0.117 Cardiac complication: *p* = 0.607 Readmission: *p* = 0.933	C	4
Zaremba et al., 2016 [30]	RCT	laparoscopic Roux-en-Y gastric bypass, laparoscopic partial vertical gastrectomy, laparoscopic sleeve, or revision of the gastric band to gastric bypass	16	29	PSG	CPAP reduced AHI and ODI by 69% (95% CI 7.3–29.8)	Logistic regression	Sleep apnea during daytime Respiratory depression from opioids	*p* = 0.002	B	2b
Kong et al., 2016 [31]	RS	laparoscopic gastric bypass, laparoscopic gastric banding, sleeve gastrectomy, open gastric bypass, retrocolic retrogastric gastrojejunostomy.	352	196	PSG	Pulmonary complications: OR 5.76 All causes complications: OR 1.88	Logistic regression	Non-CPAP group: higher rate of pneumonia, atelectasis, hypoxemia, pneumomediastinum CPAP-group: Lower rate of pneumonia, atelectasis, pneumonitis	Pulmonary complications: *p* = 0.0002 All causes complications: *p* = 0.21	B	2b
de Raaff et al., 2015 [34]	RS	laparoscopic Roux-en-Y gastric bypass, laparoscopic sleeve gastrectomy	277	1254	RP/PSG	OR 0.401	Logistic regression	Respiratory insufficiency and cardiac asthma, pneumonia, sinus tachycardia	*p* = 0.589	B	2b
Goucham et al., 2015 [32]	PS	Roux-en-Y gastric bypass, laparoscopic adjustable gastric banding, or laparoscopic sleeve gastrectomy	121	794	PSG		Logistic regression	Desaturations	*p* = 0.023 for BMI ≥ 60 and severe desaturation < 85%	B	2b
Mokhlesi et al., 2013 [35]	RS	bariatric surgery	33,196	57,832	RP/PSG	Emergent endotracheal intubation and mechanical ventilation: OR 4.35, 95% CI 3.97–4.77 CPAP/NIV: OR 14.12, 95% CI 12.09–16.51 AF: OR 1.25, 95% CI 1.11–1.41	Logistic regression	Emergent endotracheal intubation and mechanical ventilation, CPAP/NIV, and AF	*p* < 0.001	B	2b

**Table 3:** Characteristics of studies included in the analysis pertaining to orthopedic surgeries (RCT = randomized crossover trial; RS = retrospective study; PS = prospective study).

Author	Design	Type of Surgery	Sample Size		OSA Diagnosis	Effect Size	Effect Type	Complications	Significance	Oxford Level of Evidence	Grade of Recommendation
			OSA	Non-OSA							
Wong et al., 2022 [36]	RCT	hip or knee arthroplasty	234	240	home sleep apnea test (HSAT)	risk reduction 3.4%; 95% CI: −1.1% to 8.7%	Logistic regression	Delirium	*p* = 0.21	B	2b
Bai et al., 2020 [37]	RS	elective total hip or knee arthroplasty	1326	1031	STOP-BANG questionnaire/PSG	AOR 0.60, 95% CI, 0.24–1.67	Logistic regression	Respiratory depression, ICU readmission	*p* = 0.308	B	2b

**Table 4:** Characteristics of studies included in the analysis pertaining to cardiac surgeries (RCTs = randomized controlled trial; RS = retrospective study; PS = prospective study).

Author	Design	Type of Surgery	Sample Size		OSA Diagnosis	Effect Size	Effect Type	Complications	Significance	Oxford Level of Evidence	Grade of Recommendation
			OSA	Non-OSA							
Teo et al., 2024 [51]	RS	CABG	513	494	RP	HR 0.997, 95% CI: 0.994–1.000	ΔQTc change	MACCEs	*p* = 0.032	B	2b
Wolf et al., 2021 [47]	RS	CABG, isolated valve surgery or CABG with valve surgery	1555	10,450	PSG	PE: OR 2.89, 95% CI 1.11–7.52 Pneumonia: OR 1.51, 95% CI 1.14–2.02 ICU readmission: OR 1.49, 95% CI 1.17–1.90 Surgical site infection: OR 2.04, 95% CI 1.38–3.02 Renal failure: OR 1.57, 95% CI 1.09–2.27	Logistic regression	PE, pneumonia, ICU readmission, surgical site infection, renal failure, delayed extubation	PE: *p* = 0.03 Pneumonia: *p* = 0.005 ICU readmission: *p* = 0.001 Surgical site infection: *p* < 0.001 Renal failure: *p* = 0.015	B	2b
Hunt et al., 2022 [54]	RCTs open-label parallel-group	Cryoballoon PVI, RF ablation	243	336	RP	OR 1.0, 95% CI 0.42–2.4	Logistic regression	AF, pericardical tamponade, cerebral ischemic event	AF: *p* = 1.00	B	2b
Guo et al., 2021 [44]	PS	CABG	142	36	PSG	eGFR: OR 0.94, 95% CI 0.89–0.99 AHI: OR 1.07, 95% CI 1.01–1.13	Logistic regression	AKI requiring dialysis, prolonged postoperative ventilation time	AKI requiring dialysis: *p* = 0.02 Prolonged postoperative ventilation time: *p* = 0.05	B	2b
Tafelmeier et al., 2018 [46]	RS	CABG	23	77	RP	Prolonged LOS > 9 days: OR 3.34, 95% CI 1.24–9.01 Prolonged need of vasopressors > 48 h: OR 2.94, 95% CI 1.05–8.23	Logistic regression	Prolonged LOS > 9 days, prolonged need of vasopressors > 48 h, tracheostomy requirement	Prolonged LOS > 9 days: *p* = 0.017 Prolonged need of vasopressors > 48 h: *p* = 0.04 Tracheostomy: *p* = 0.028	B	2b
Kaw et al., 2017 [57]	RS	CABG and/or valve replacement	132	58	PSG	BMI > 32 kg/m^2^ had 15% increased odds of AF: OR = 1.15, 95% CI 1.05–1.26	Logistic regression	AF	*p* < 0.003	B	2b
Rupprecht et al., 2017 [48]	PS	CABG	151	68	RP	Respiratory complications: OR 2.40, 95% CI 1.15–4.97 Cardiac complications: OR 1.75, 95% CI 0.93–3.27 Higher risk of sepsis: OR 2.24, 95% CI 0.71–7.02	Logistic regression	Higher 30-day mortality, nonspecific desaturation events, acute hypoxemia due to pneumonia, sepsis and septic shock, supraventricular arrhythmias, sopor and coma, AKI	Respiratory complications: *p* = 0.02 Cardiac complications: *p* = 0.08 Higher risk of sepsis: *p* = 0.17	B	2b
Ding et al., 2016 [42]	PS	Valve replacement	54	236	PSG	Longer ICU stay: OR 2.318, 95% CI 1.241–4.329 Mechanical ventilation: OR 2.050, 95% CI 1.028–4.085 Pacemaker use: OR 2.477, 95% CI 1.196–5.131	Logistic regression	Longer ICU stay, prolonged mechanical ventilation time (≥20 h), respiratory insufficiency, and pacemaker requirement	Longer ICU stay: *p* = 0.008 Mechanical ventilation: *p* = 0.041 Pacemaker use: *p* = 0.015	B	2b
Lee et al., 2016 [52]	PS	PCI	594	717	RP	HR 1.57, 95% CI 1.10–2.24	Logistic regression	MACCEs	*p* = 0.013	B	2b
Kua et al., 2016 [45]	PS	CABG	75	75	Sleep study	OR 2.89, 95% CI 1.09–7.09	Logistic regression	AKI	*p* = 0.03	B	2b
Uchôa et al., 2015 [40]	PS	CABG	37	30	PSG	MACCEs: OR 4.10 95% CI 1.94–385.24 New revascularization: OR 2.02 95% CI 1.21–64.22 Typical angina: OR 10.05 95% CI 1.12–62.25 AF: OR 12.56 95% CI 1.44–159.21	Logistic regression	MACCEs, new revascularization, typical angina and AF	MACCEs: *p* = 0.004 New revascularization: *p* = 0.01 Typical angina: *p* = 0.02 AF: *p* = 0.006	B	2b
Szymanski et al., 2015 [55]	PS	catheter ablation of AF	114	137	RP	OR 2.58 95% CI 1.52–4.38	Logistic regression	AF recurrence	*p* < 0.0001	B	2b
Zhao et al., 2015 [41]	PS	CABG	69	69	PSG	OR 4.63 95% CI: 1.24–17.31	Logistic regression	Readmissions due to cardiovascular events	*p* = 0.023	B	2b
Wu et al., 2015 [50]	RS	PCI	390		RP/PSG	HR 2.13 95% CI 1.19–3.81	Logistic regression	Repeat revascularization	*p* = 0.011	B	2b
Foldvary-Schaefer et al., 2015 [58]	RS	CABG, single valve repair/replacement, CABG and single valve repair/re-placement or >2 valve repair/replacement, or others, including septal myomectomy, right atrial mass removal, cardiac catheterization, and cardioverter defibrillator placement.	51	56	PSG		Logistic regression	OR tube time, total tube time, ICU LOS, ICU readmission, insulin infusion in ICU, readmission by 30 days, prolonged intubation, respiratory failure, reintubation, hypoxemia, tracheostomy, myocardial infarction, arrhythmia, encephalopathy, infection, death	*p* > 0.05	B	2b
Utriainen et al., 2014 [49]	PS	elective sub-inguinal revascularisation	39	45	PSG	HR 4.4 95% CI 1.8–10.6	Logistic regression	MACCEs	*p* = 0.001	B	2b
Roggenbach et al., 2014 [43]	PS	elective coronary artery surgery or heart valve replacement/repair, either with or without coronary bypass grafting	83	9	RP	OR 6.4 95% CI 2.6–15.4	Logistic regression	Delirium	*p* < 0.001	B	2b
van Oosten et al., 2014 [38]	PS	CABG	132	145	modified Berlin questionnaire/PSG	AF: OR 2.18 95% CI 1.30–3.65	Logistic regression	AF, reintubation, postoperative atrial flutter, other postoperative arrhythmias, LOS	AF: *p* = 0.003	B	2b
Neilan et al., 2013 [56]	PS	PVI	142	578	RP	HR 1.61, 95% CI 1.35–1.92	Logistic regression	AF recurrence	*p* < 0.001	B	2b
Fein et al., 2013 [39]	RS	PVI	62	324	PSG	PVI(+)OSA(+)CPAP(+): HR 0.7 95% CI 0.3–1.59 PVI(+)OSA(+)CPAP(−): HR 2.15 95% CI 1.10–5.44	Logistic regression	AF recurrence	PVI(+)OSA(+)CPAP(+): *p* = 0.39 PVI(+)OSA(+)CPAP(−): *p* = 0.02	B	2b

**Table 5:** Characteristics of studies included in the analysis pertaining to otorhinolaryngologic surgeries (RS = retrospective study).

Author	Design	Type of Surgery	Sample Size		OSA Diagnosis	Effect Size	Effect Type	Complications	Significance	Oxford Level of Evidence	Grade of Recommendation
			OSA	Non-OSA							
Passeri LA et al., 2016 [59]	RS	single piece Le Fort I osteotomy, bilateral sagittal split mandibular osteotomies and either a genial tubercle advancement or genioplasty	28	26	PSG	RR of complications OSA vs. DFD = 3.04 RR for major complications = 10.75	Descriptive & comparative stats	Dysesthesia, infection, hardware removal, reoperation	*p* = 0.003	B	2b
Kandasamy T et al., 2013 [60]	RS	standard Fujita type UPPP (with or without tonsillectomy) is performed with cautery	345		PSG	AHI ≥ 22: OR 2.21, 95% CI 1.166–4.188 BMI ≥ 30: OR 2.70, 95% CI 1.48–4.91 both an AHI ≥ 22 and a BMI ≥ 30: OR 3.48, 95% CI 1.56–7.78	Logistic regression	Oxyhemoglobin desaturation	*p* < 0.05	B	2b

## Data Availability

The data extracted from the included studies used in this review are available from the corresponding author upon reasonable request. No analytic code or additional materials were used beyond what is reported in the manuscript.
